# Prescription Fills for Semaglutide Products by Payment Method

**DOI:** 10.1001/jamahealthforum.2024.2026

**Published:** 2024-08-02

**Authors:** Christopher Scannell, John Romley, Rebecca Myerson, Dana Goldman, Dima M. Qato

**Affiliations:** 1 Schaeffer Center for Health Policy & Economics, University of Southern California, Los Angeles; 2 Los Angeles Area Health Service Research Training Program, Fielding School of Public Health, University of California, Los Angeles; 3 Price School of Public Policy, University of Southern California, Los Angeles; 4 Program on Medicines and Public Health, Alfred E. Mann School of Pharmacy and Pharmaceutical Sciences, University of Southern California, Los Angeles; 5 Department of Population Health Sciences, University of Wisconsin, Madison

## Abstract

This cross-sectional study examines trends in US prescription fills for semaglutide products by payment method between January 2021 and December 2023.

## Introduction

Semaglutide was first approved under the brand name Ozempic (Novo Nordisk) as a weekly injection by the US Food and Drug Administration (FDA) in 2017 and is a first-line treatment for patients with type 2 diabetes at high cardiovascular risk.^1^ Alongside evidence of semaglutide’s cardiovascular and weight loss benefits,^2^ the FDA approved Rybelsus (Novo Nordisk), an oral formulation for the treatment of type 2 diabetes, in 2019, and Wegovy (Novo Nordisk), another injectable formulation for obesity, in June 2021.^1^ Increased awareness of semaglutide’s weight loss benefits has fueled demand, including for off-label use, contributing to shortages for Ozempic and Wegovy since March 2022.^3^ Semaglutide’s limited supply and insurance coverage, including coverage prohibitions for weight loss drugs in Medicare Part D and coverage restrictions (prior authorization, nonpreferred status, or step therapy) in many state Medicaid plans,^4,5^ may contribute to barriers and disparities in accessing semaglutide due to its high out-of-pocket costs.^6^ In this cross-sectional study, we analyze trends in prescriptions dispensed at retail pharmacies for semaglutide between January 2021 and December 2023.

## Methods

We used data from IQVIA’s National Prescription Audit PayerTrak, which captures 92% of prescriptions filled and dispensed to individuals at retail pharmacies in the US. We calculated monthly fills for semaglutide by drug brand (Ozempic, Wegovy, and Rybelsus) and payment method (commercial insurance, Medicaid, Medicare Part D, and cash) between January 2021 and December 2023. This study was exempted and informed consent was waived because it is not considered human participants research by the University of Southern California institutional review board. The study followed the STROBE reporting guideline.

Analyses were conducted using Stata, version 17.0 (StataCorp, LLC). A 2-sided *P* < .05 was considered significant.

## Results

The number of semaglutide fills increased by 442% between January 2021 and December 2023 (from 471 876 to 2 555 308), with Ozempic accounting for over 70% or more of semaglutide fills during this period (Figure). Although increases were observed for all 3 drug brands, the extent and timing of these increases varied. For example, Ozempic peaked in August 2023 (1 968 892 fills) before reaching a plateau, increasing by 392% between January 2021 and December 2023. Notable increases in Wegovy fills started in January 2023 (157 554 fills), peaked in May 2023 (519 510 fills), and subsequently plateaued, increasing by more than 1361% between July 2021 and December 2023.

**Figure.**
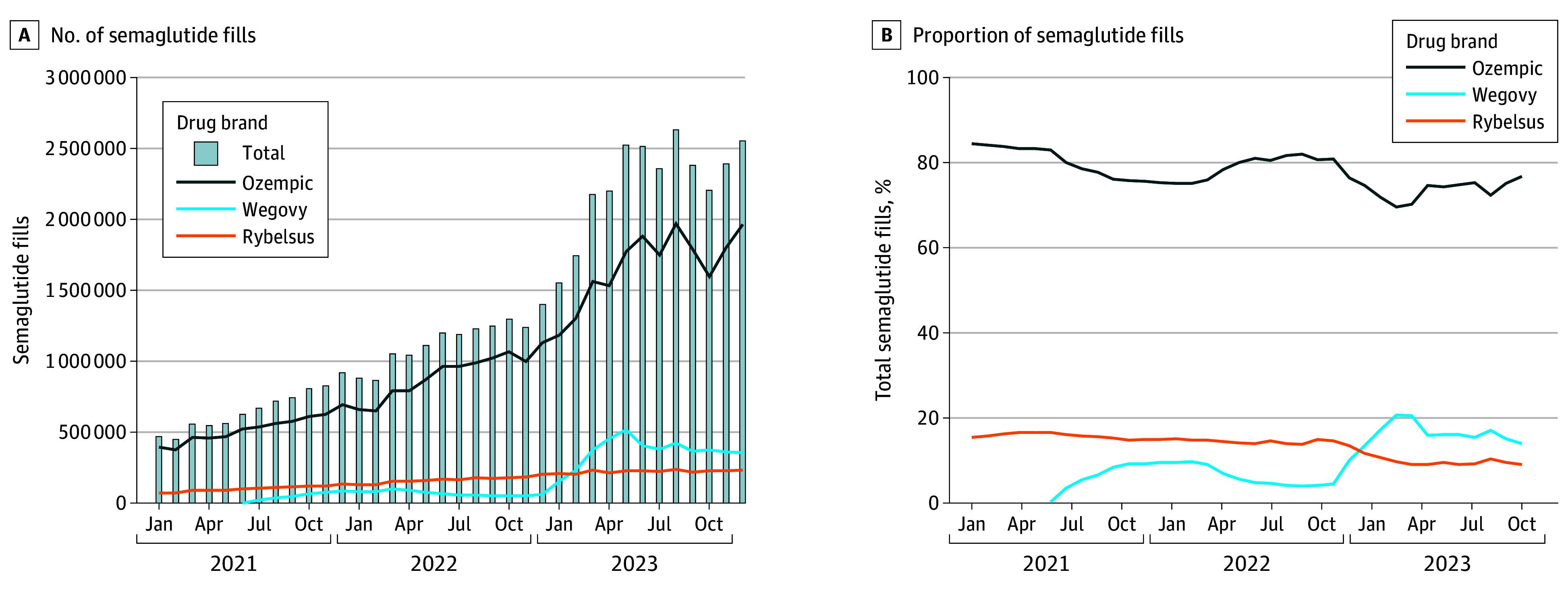
Trends in Semaglutide Prescription Fills by Drug Brand Between January 2021 and December 2023 These data are from IQVIA’s National Prescription Audit PayerTrak on monthly prescription fills dispensed at retail pharmacies in the US.

Increases in monthly semaglutide fills were observed across all payment methods for all 3 drug brands between 2021 and 2023 (Table). However, semaglutide prescriptions paid through commercial insurance persistently accounted for most fills for all 3 drug brands, particularly for Wegovy.

**Table.** Changes in Semaglutide Prescriptions Filled by Drug Brand and Method of Payment Between January 2021 and December 2023^a^

Characteristic	Fills by method of payment							
	No. of monthly fills, mean (SD)				Proportion of monthly fills, mean (IQR)			
	2021	2022	2023	*P* value^b^	2021	2022	2023	*P* value^b^
All semaglutide fills	659 492 (147 800)	1 148 123 (161 532)	2 270 564 (326 866)	<.001	NA	NA	NA	NA
**Ozempic**								
Total	526 289 (95 463)	909 927 (154 356)	1 676 258 (246 594)	<.001	NA	NA	NA	NA
Commercial	346 850 (66 124)	598 503 (104 237)	1 027 892 (143 899)	<.001	65.8 (65.2-66.3)	65.7 (65.2-66.2)	61.4 (60.7-62.2)	<.001
Medicaid	40 096 (6371)	75 385 (15 834)	163 142 (26 309)	<.001	7.6 (7.5-7.9)	8.2 (7.9-8.6)	9.7 (9.5-10.0)	<.001
Medicare Part D	138 327 (22 854)	233 028 (33 529)	478 492 (78 055)	<.001	26.4 (26.0-26.7)	25.7 (25.2-26.4)	28.5 (27.8-29.3)	<.001
Cash	1017 (195)	3011 (1255)	6732 (292)	<.001	0.2 (0.2-0.2)	0.3 (0.2-0.4)	0.4 (0.4-0.4)	<.001
**Wegovy**								
Total	49 599 (30 209)	70 958 (17 777)	368 686 (94 174)	<.001	NA	NA	NA	NA
Commercial	43 501 (27 411)	65 761 (16 149)	329 340 (82 461)	<.001	86.0 (83.8-89.4)	92.8 (92.4-93.4)	89.5 (89.1-89.7)	.03
Medicaid	363 (351)	2483 (649)	30 797 (9721)	<.001	0.6 (0.3-0.9)	3.6 (3.4-4.2)	8.2 (8.2-8.5)	<.001
Medicare Part D	324 (187)	700 (151)	4438 (1785)	<.001	0.8 (0.6-0.7)	1.0 (0.9-1.1)	1.2 (0.9-1.5)	.02
Cash	5410 (2583)	2014 (1593)	4112 (689)	.24	12.7 (9.0-15.2)	2.6 (1.6-3.1)	1.2 (1.0-1.2)	<.001
**Rybelsus**								
Total	104 270 (20 656)	167 238 (21 206)	225 620 (10 501)	<.001	NA	NA	NA	NA
Commercial	73 242 (12 415)	106 716 (12 283)	130 991 (5390)	<.001	70.7 (68.7-72.2)	63.9 (63.1-64.5)	58.1 (57.6-58.4)	<.001
Medicaid	4696 (1557)	12 544 (2856)	19 687 (835)	<.001	4.4 (3.9-5.0)	7.4 (6.9-8.1)	8.7 (8.6-9.0)	<.001
Medicare Part D	25 952 (6926)	47 715 (6103)	74 311 (4683)	<.001	24.5 (23.4-26.2)	28.5 (28.3-28.7)	32.9 (32.4-33.6)	<.001
Cash	380 (226)	263 (42)	631 (220)	.01	0.4 (0.2-0.6)	0.2 (0.1-0.2)	0.3 (0.2-0.3)	.20

Abbreviation: NA, not applicable.

^a^ These data are from IQVIA’s National Prescription Audit PayerTrak on monthly prescription fills dispensed at retail pharmacies in the US.

^b^ *P* values represent the level of significance using a *t* test for the difference between 2023 and 2021.

In 2023, commercial insurance accounted for 61.4% (IQR, 60.7%-62.2%) of Ozempic, 89.5% (IQR, 89.1%-89.7%) of Wegovy, and 58.1% (IQR, 57.6%-58.4%) of Rybelsus fills. While Medicare Part D accounted for 28.5% (IQR, 27.8%-29.3%) and 32.9% (IQR, 32.4%-33.6%) of Ozempic and Rybelsus fills, respectively, in 2023, it only accounted for 1.2% (IQR, 0.9%-1.5%) of Wegovy fills. In contrast, Medicaid accounted for less than 10% of semaglutide fills for all 3 drug brands in 2023.

## Discussion

The number of prescriptions filled for semaglutide has increased substantially, reaching 2.6 million prescriptions filled at retail pharmacies by December 2023. While Ozempic persistently accounted for most semaglutide fills, increases were considerably greater for Wegovy since its approval for weight loss in June 2021. These increases, which primarily occurred following increased awareness of weight-loss benefits in late 2022,^3^ are likely contributing to the FDA-reported shortage of Ozempic and Wegovy first issued in March 2022. Despite the disproportionate burden of obesity in Medicaid and Medicare Part D populations^4,5^ and recent increases in public spending on weight-loss medications,^4,5,6^ most Wegovy fills were for the commercially insured. Limitations of this study include a lack of data on individual-level variables (age, race, and ethnicity) and indications for use (diabetes or obesity). Future research should examine how changes in Medicare Part D and Medicaid coverage restrictions influence disparities in access to these essential medications.

## Notes

Supplement.
Data Sharing Statement
