# Supplementary material for: Prescription Fills for Semaglutide Products by Payment Method
Source: JAMA Health Forum. 2024 Aug 2;5(8):e242026. doi: 10.1001/jamahealthforum.2024.2026 (PMC11297375; doi:10.1001/jamahealthforum.2024.2026)
Supplement: Supplement. — Data Sharing Statement [file jamahealthforum-e242026-s001.pdf]

Data Sharing Statement

Scannell. Prescription Fills for Semaglutide Products by Payment Method. *JAMA Health Forum*. Published August 02, 2024. doi:10.1001/jamahealthforum.2024.2026

Data
Data available: No
